# Supplementary material for: Increased incompatibility of heterologous algal symbionts under thermal stress in the cnidarian-dinoflagellate model Aiptasia
Source: Commun Biol. 2022 Jul 28;5:760. doi: 10.1038/s42003-022-03724-y (PMC9334593; doi:10.1038/s42003-022-03724-y)
Supplement: Supplementary file 2 — Supplementary Information [file 42003_2022_3724_MOESM2_ESM.pdf]

Figure S1: Differential expressed genes shared among Aiptasia strains CC7, CC7-B01 and H2.

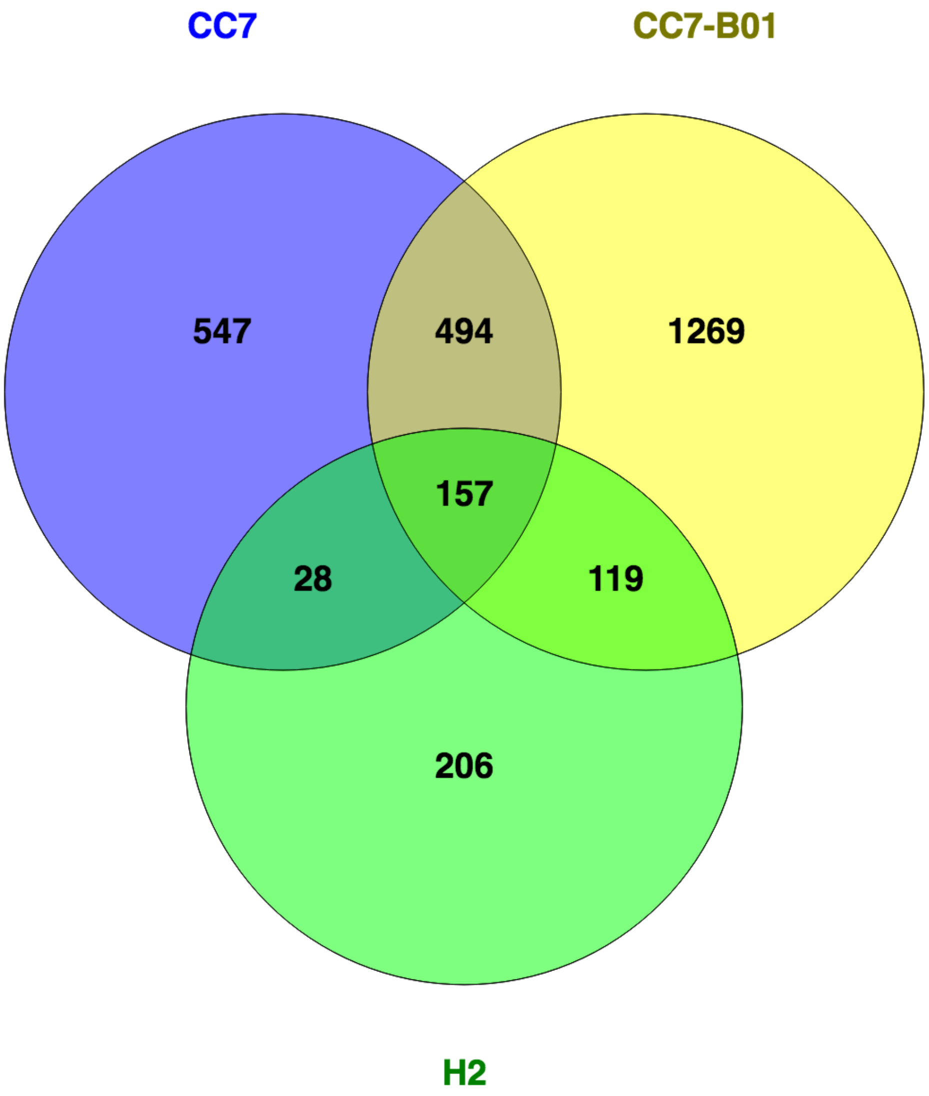

Few genes are commonly expressed among all the strains responses to heat stress. The largest overlap is observed between CC7 and CC7-B01, followed by CC7-B01 and H2. The two homologous strains, CC7 and H2, share the least number of genes.
