# Supplementary material for: Increased incompatibility of heterologous algal symbionts under thermal stress in the cnidarian-dinoflagellate model Aiptasia
Source: Commun Biol. 2022 Jul 28;5:760. doi: 10.1038/s42003-022-03724-y (PMC9334593; doi:10.1038/s42003-022-03724-y)
Supplement: Supplementary file 3 — Description of Additional Supplementary Files [file 42003_2022_3724_MOESM3_ESM.pdf]

Description of Additional Supplementary Files

File name: Supplementary Data 1
Description: Differentially expressed genes in Aiptasia strains CC7, H2 and CC7-B01 after heat stress.

File name: Supplementary Data 2
Description: GO-term analysis output for differentially expressed genes in Aiptasia strains CC7, H2 and CC7-B01.

File name: Supplementary Data 3
Description: Symbiosis genes (according to Cui et al., 2018) expression in CC7, H2 and CC7-B01.

File name: Supplementary Data 4
Description: Caspase activity.
